# UVA-Radiation Exposure of Different Durations Promoted the Growth, Phytochemicals and Glucosinolate Biosynthesis of Chinese Kale

**DOI:** 10.3390/ijms23147619

**Published:** 2022-07-10

**Authors:** Meifang Gao, Yamin Li, Haozhao Jiang, Rui He, Rui Shi, Shiwei Song, Houcheng Liu

**Affiliations:** College of Horticulture, South China Agricultural University, Guangzhou 510642, China; gmf@stu.scau.edu.cn (M.G.); yaminli@stu.scau.edu.cn (Y.L.); jhzh111@stu.scau.edu.cn (H.J.); ruihe@stu.scau.edu.cn (R.H.); ruishi@stu.scau.edu.cn (R.S.); swsong@scau.edu.cn (S.S.)

**Keywords:** UVA, Chinese kale, growth, glucosinolate

## Abstract

Ultraviolet-A (UVA) (315–400 nm) is an essential environmental signal that regulates plant development and affects phytochemicals biosynthesis, including glucosinolate biosynthesis. The effects of different UVA (380 ± 10 nm, 40 μmol/m^2^/s) exposure durations, including 0 h/d (UV0), 6 h/d (UV6) and 12 h/d (UV12), on the growth and phytochemicals of Chinese kale (*Brassica alboglabra*) under white 250 μmol/m^2^/s LEDs were investigated. UVA exposure of different durations influenced the growth and phytochemicals biosynthesis of Chinese kale. Prolonging UVA irradiation throughout the growth cycle positively affected the growth and the development of Chinese kale, with evident increases in the dry weights of shoots and roots, plant height, stem diameter, specific leaf weight and flower budding rate. The application of UVA increased the soluble sugar content, whereas higher flavonoid content and antioxidant capacity (FRAP) and lower nitrate content were only observed in Chinese kale exposed to UV6 treatment. Besides, the qPCR assay showed that supplemental UVA-radiation exposure up-regulated the gene expressions of *UVR8*, transcription factors genes and genes related to the glucosinolate biosynthesis pathway, thereby promoting the accumulation of glucosinolates. Therefore, supplemental UVA-radiation exposure for 12 h/d was more conducive to plant growth, while supplemental UVA-radiation exposure for 6 h/d was better for phytochemical biosynthesis in Chinese kale in an artificial-light plant factory.

## 1. Introduction

Chinese kale (*Brassica alboglabra*), one of the important cruciferous vegetables, is widespread in South China and Southeast Asia [1]. People popularly consume its flower stalk and tender leaves due to its good flavor, vivid green color and abundance in antioxidant and anti-cancer compounds, including glucosinolates, vitamin C, flavonoids and polyphenols [2]. Particularly, glucosinolates are critical secondary metabolites containing nitrogen and sulfur in cruciferous plants [1]. In addition to endowing plants with special flavor, glucosinolates and their hydrolysis products perform important biological activities. They play a defensive role in plants and in the human body [3].

Light is one of the important factors regulating plant growth and development. The spectral component of solar radiation effective for plant photosynthesis is photosynthetically active radiation (PAR; 400–700 nm). Although UVA does not belong to PAR and it accounts for only a small proportion of the radiation reaching the Earth’s surface [4], it can substantially affect plant growth and development as well as plant metabolic responses, as it accounts for more than 90% of the UV radiation reaching the Earth [5]. There have been many studies on the effects of UVA (wavelength and intensity) [6,7,8,9,10,11] and of supplemental UVA exposures (treatment period and duration, etc.) [5,6,12] on the biomass and nutritional quality of different vegetables. Exposed to UVA, plants can induce the biosynthesis of glucosinolates (GSLs). Previous research on pak choi (*Brassica campestris* L. ssp. *chinensis* var. *communis*) [10,11], broccoli (*Brassica oleracea* var. *italica*) [13,14] and Brussels sprouts (*Brassica oleracea* var. *gemmifera*) [15], as well as analyses of different UVA wavelengths and dose treatments of Chinese kale (*Brassica alboglabra*) [11], revealed that targeted UVA irradiation could enhance the accumulation of glucosinolates. However, the effects of UVA-radiation exposure of different durations on the growth and development of Chinese kale remain relatively unknown.

In plants, the glucosinolate biosynthetic pathways includes side-chain elongation, core structure formation and side-chain modification. Regulatory transcription factors (TFs) also contribute to the production of diverse glucosinolate components. This pathway was thoroughly characterized in *Arabidopsis thaliana* with the genes encoding the following glucosinolate biosynthetic enzymes: branched-chain amino acid aminotransferase 4 (BCAT4), methylthioalkylmalate synthase enzymes (MAM1 and MAM3), isopropylmate isomerases (IIL1, IPM1 and IPM2), isopropylmalate dehydrogenases (IMDH1, IMDH2 and IMDH3), cytochrome P450 enzyme, glutathione S-transferases (GSTFs), γ-Glutamyl peptidase 1 (GGP1), C-S lyase (SUR1), UDP-glycosyltransferase 74 (UGT74) family, sulfotransferases (SOTs), five flavin monooxygenases (FMO_GS-OX_), 2-oxoglutarate-dependent dioxygenases (AOPs), indole glucosinolate O-methyltras-ferases (IGMTs), etc. However, the molecular pathways underlying UVA-induced glucosinolate accumulation in Chinese kale have not been elucidated [16].

Four types of photoreceptors have been found in plants, namely, phytochromes (PHYs; the receptors of red and far-red wavelengths), cryptochromes (CRYs; the receptors of UVA and blue wavelengths), phototropins (PHOTs; the receptors of UVA and blue wavelengths) and UV RESISTANCE LOCUS8 (UVR8; the receptor of UVB wavelengths). These photoreceptors have some relationships with the expressions of glucosinolate biosynthesis genes. In broccoli sprouts subjected to UVB irradiation, UVR8 interacted with downstream COP1 to activate downstream transcription factor HY5 and then regulated the expressions of key genes such as *FMO_GS-OX5_* and *CYP81F2*, inducing higher accumulations of 4-methylsulfinylbutyl glucosinolate and 4-methoxy-indol-3-ylmethyl glucosinolate [17]. When subjected to low red:far-red light ratios (0.55) of irradiation in *Arabidopsis thaliana*, photoreceptor PHYB became physiologically inactivated, while the activity of HY5 was inhibited, and the expressions of glucosinolate biosynthesis genes (*SUR2*, *CYP79B2*, *CYP79B3* and *CYP83A1*) decreased, resulting in reduced contents of indol-3-ylmethyl glucosinolate [18].

In this study, the growth and phytochemicals biosynthesis in Chinese kale subjected to UVA treatments of different lengths in an artificial-light plant factory were evaluated to clarify the effects of UVA exposure duration on growth, glucosinolate metabolism and regulatory mechanisms in Chinese kale development.

## 2. Results

### 2.1. Growth Properties

**Figure 1:**
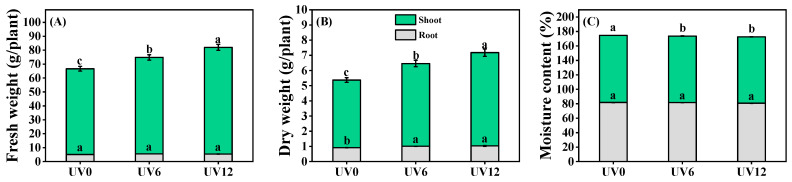
Growth properties of Chinese kale exposed to UVA-radiation exposure of different durations. (**A**) Fresh weights of shoots and roots (S-FW and R-FW). (**B**) Dry weights of shoots and roots (S-DW and R-DW). (**C**) Moisture contents of shoots and roots (S-MC and R-MC). Different lowercase letters (a–c) labeled on the vertical bar (means ± standard error, *n* = 8) represent significant differences by one-way ANOVA with multiple-range tests (*p* ≤ 0.05).

The growth properties of Chinese kale were significantly affected by different lengths of UVA radiation exposure. Chinese kale subjected to supplemental UVA treatments (UV6 and UV12) exhibited higher fresh and dry weights of shoots and roots and lower shoot moisture contents than plants exposed to UV0 (Figure 1). Specifically, compared with UV0, shoot fresh weights significantly increased by 9.94% and 15.86% (Figure 1A) and shoot dry weights by 23.92% and 31.92% (Figure 1B), respectively, when plants were subjected to UV6 and UV12 treatments. In addition, root dry weight was also significantly higher, by 1.12 times, in Chinese kale exposed to both UVA treatments (UV6 and UV12) in comparison with UV0 (Figure 1B). UV6 and UV12 did not affect the root moisture contents in Chinese kale but significantly decreased the shoot moisture contents, presenting reductions of 0.84% and 1.08%, respectively, when compared with UV0 (Figure 1C).

### 2.2. Morphology

**Figure 2:**
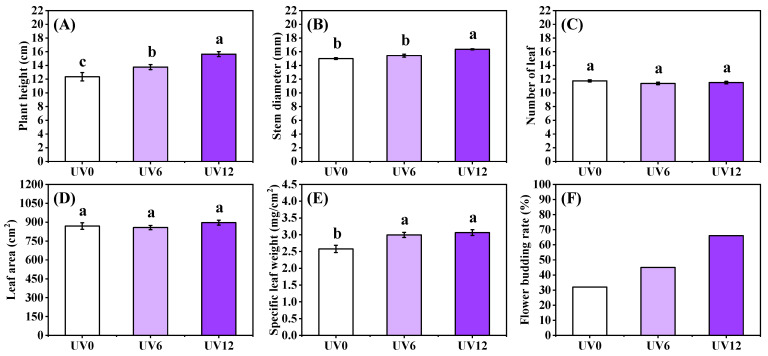
Morphology of Chinese kale exposed to different durations of UVA-radiation exposure. (**A**) Plant height (PH). (**B**) Stem diameter (SD). (**C**) Number of leaves (LN). (**D**) Leaf area (LA). (**E**) Specific leaf weight (SLW). (**F**) Flower budding rate. Different lowercase letters (a–c) labeled on the vertical bar (means ± standard error, *n* = 8) represent significant differences by one-way ANOVA with multiple-range tests (*p* ≤ 0.05).

Supplemental UVA exposure induced remarkable changes in morphological responses such as plant height, stem diameter, specific leaf weight (an index of leaf thickness) and flower budding rate. Concretely, compared with UV0, plant height was significantly higher, by 1.11 and 1.27 times, in plants exposed to UV6 and UV12 treatments, respectively (Figure 2A), and the stem diameter of plants treated with UV12 was 10.07% higher (Figure 2B). Significant increases were observed in the specific leaf weight of plants treated with UV6 and UV12, but there were no statistically significant differences between the plants subjected to the two UVA treatments (Figure 2E). Regarding the flower budding rate, there was an increasing trend with the increase in the duration of supplemental UVA exposure, and the maximum flower budding rate of 45-day-old Chinese kale was 66% (UV12) (Figure 2F). However, no significant differences were observed in the number of leaves or in the leaf area of plants treated with UV6 and UV12 (Figure 2C,D).

### 2.3. Leaf Pigment Contents

**Figure 3:**
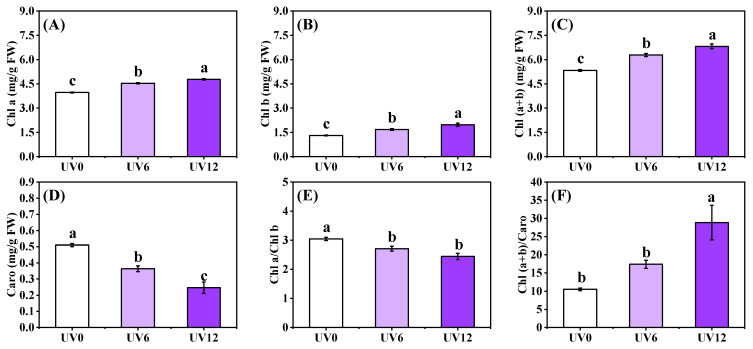
Leaf pigment content of Chinese kale subjected to different durations of UVA-radiation exposure. (**A**) Chl a. (**B**) Chl b. (**C**) Chl (a + b). (**D**) Caro. (**E**) Chl a/Chl b. (**F**) Chl (a + b)/Caro. Different lowercase letters (a–c) labeled on the vertical bar (means ± standard error, *n* = 3) represent significant differences by one-way ANOVA with multiple-range tests (*p* ≤ 0.05).

By prolonging UVA exposure, the contents of Chl a, Chl b and Chl (a + b) in Chinese kale leaf increased (Figure 3A–C), whereas Caro content decreased (Figure 3D). Compared with UV0, significant reductions were observed in Chl a/Chl b of plants treated with UV6 and UV12 (Figure 3E), whereas with the increase in the duration of supplemental UVA exposure, an increasing trend of Chl (a + b)/Caro contents and a significant difference were detected in plants subjected to UV12 treatment (Figure 3F).

### 2.4. ChlF Properties

**Figure 4:**
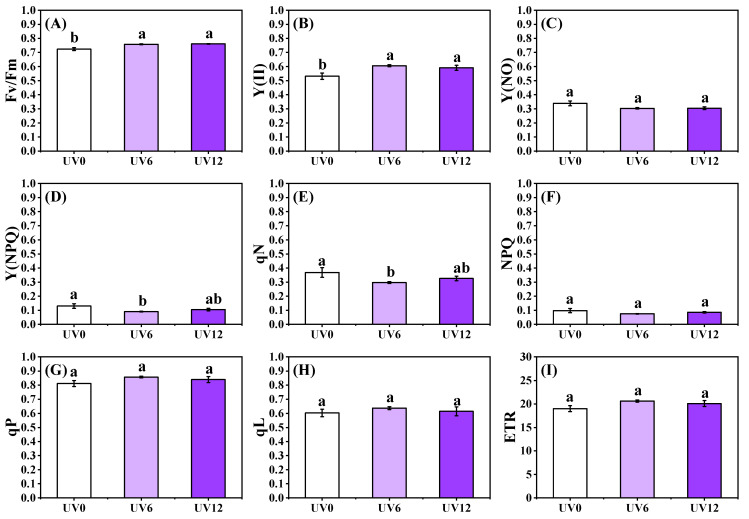
ChlF properties of Chinese kale subjected to different durations of UVA-radiation exposure. (**A**) Fv/Fm. (**B**) Y(II). (**C**) Y(NO). (**D**) Y(NPQ). (**E**) qN. (**F**) NPQ. (**G**) qP. (**H**) qL. (**I**) ETR. Different lowercase letters (a,b) labeled on the vertical bar (means ± standard error, *n* = 3) represent significant differences by one-way ANOVA with multiple-range tests (*p* ≤ 0.05).

Obviously greater Fv/Fm and Y(II) were observed in plants treated with UV6 and UV12 than in those treated with UV0, whereas no significant differences were observed between UV6 and UV12 (Figure 4A,B). Interestingly, significant reductions in Y(NPQ) and qN were only found in plants subjected to UV6 treatment and were significantly lower, by 30.42% and 19.35%, than those in plants treated with UV0, respectively (Figure 4D,E). However, no significant differences were observed in Y(NO), NPQ, qP, qL and ETR in Chinese kale treated with UV6 and UV12 (Figure 4C,F–I).

### 2.5. Shoot Phytochemical Contents

**Figure 5:**
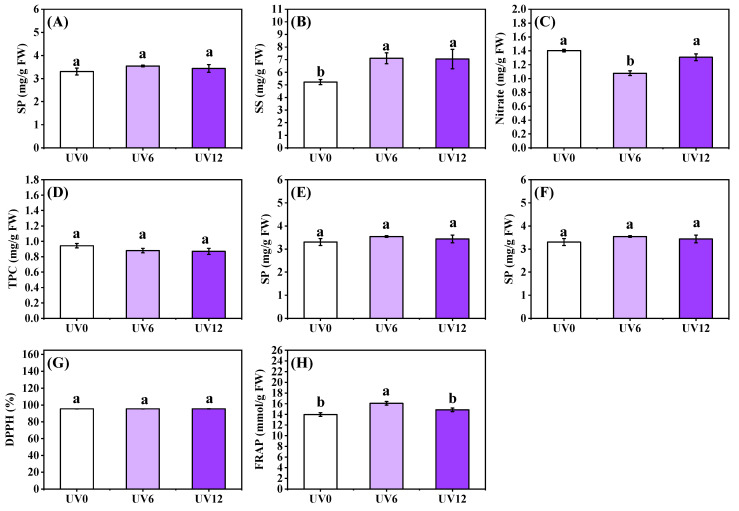
Shoot phytochemical contents of Chinese kale subjected to different durations of UVA-radiation exposure. (**A**) SP. (**B**) SS. (**C**) Nitrate. (**D**) VC. (**E**) TF. (**F**) TPC. (**G**) DPPH. (**H**) FRAP. Different lowercase letters (a,b) labeled on the vertical bar (means ± standard error, *n* = 3) represent significant differences by one-way ANOVA with multiple-range tests (*p* ≤ 0.05).

The shoot phytochemical contents of Chinese kale were significantly affected by UVA radiation (Figure 5). Compared with UV0, UV6 and UV12 notably increased SS content by 36.08% and 35.05%, respectively, but there were no significant differences between UV6 and UV12 (Figure 5B). Interestingly, UV6 treatment observably reduced the nitrate content of Chinese kale by 23.27% compared with UV0 (Figure 5C). The highest TF content was observed with UV6 treatment, followed by Chinese kale subjected to UV12 treatment (Figure 5E). In addition, FRAP followed a trend similar to that of TF content, but there were no statistically significant differences in FRAP between UV0 and UV12 (Figure 5H). However, no significant differences were detected in the contents of SP, VC, TPC and DPPH among UV0, UV6 and UV12 treatments (Figure 5A,D,F,G).

### 2.6. Mineral Element Accumulation

**Figure 6:**
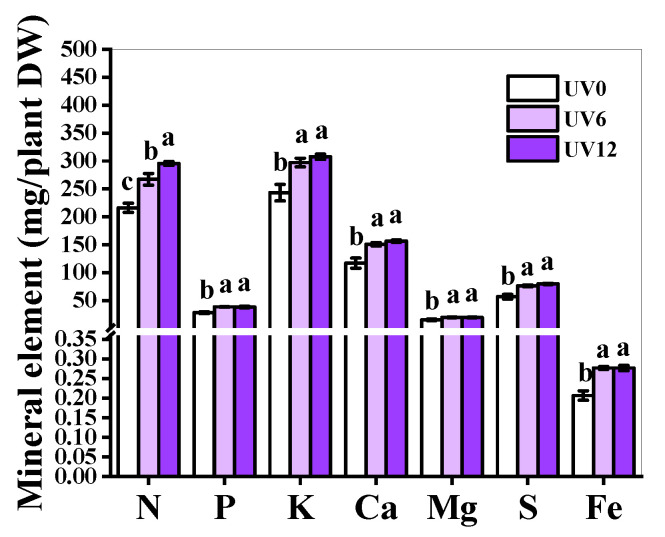
Mineral element accumulations of Chinese kale subjected to different durations of UVA-radiation exposure. Different lowercase letters (a–c) labeled on the vertical bar (means ± standard error, *n* = 3) represent significant differences by one-way ANOVA with multiple-range tests (*p* ≤ 0.05).

There were many enhancements of the accumulation of mineral elements in the UV6 and UV12 treatment groups (Figure 6). Concretely, N accumulation was distinctively boosted by UV12, followed by UV6, in comparison with UV0. Additionally, the accumulation of P, K, Ca, Mg, S and Fe was increased in plants treated with UV6 and UV12, without a noticeable difference between the two treatments (Figure 6).

### 2.7. Glucosinolate Profiles

**Figure 7:**
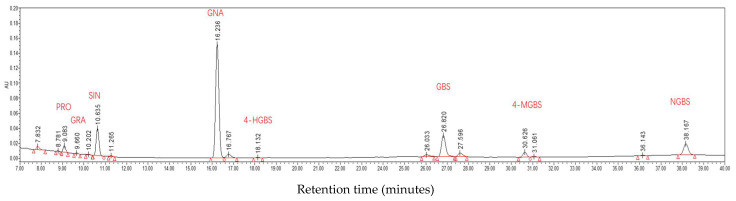
The composition of GSLs in Chinese kale. PRO (Progoitrin), GRA (Glucoraphanin), SIN (Sinigrin), GNA (gluconapin), 4-HGBS (4-hydroxy-glucobrassicin), GBS (Glucobrassicin), 4-MGBS (4-methoxy-glucobrassicin) and NGBS (Neoglucobrassicin).

**Figure 8:**
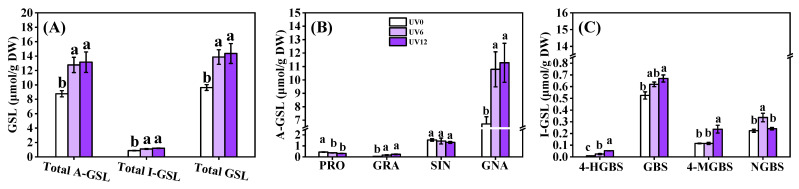
Glucosinolate profiles of Chinese kale subjected to different durations of UVA-radiation exposure. (**A**) GSL (glucosinolate). (**B**) A-GSL (aliphatic glucosinolate). (**C**) I-GSL (indolic glucosinolate). Different lowercase letters (a–c) labeled on the vertical bar (means ± standard error, *n* = 3) represent significant differences by one-way ANOVA with multiple-range tests (*p* ≤ 0.05).

Eight individual GSLs were identified in Chinese kale (Figure 7), including four aliphatic GSLs (PRO, GRA, SIN and GNA) and four indolic GSLs (4-HGBS, GBS, 4-MGBS and NGBS). The total aliphatic GSLs accounted for approximately 90% of the total GSLs (the eight GSLs). The obviously increased contents of aliphatic GSLs (45.84% and 50.19%) and indolic GSLs (25.73% and 37.57%) in plants treated with UV6 and UV12 accounted for the significant increase in total GSL content (44.02% and 49.04%) (Figure 8A). Concerning the individual GSLs, GNA was predominant in Chinese kale (Figure 8B). Most of the individual GSL contents were significantly enhanced by UV6 (60.58–217.64%) and UV12 (27.56–462.37%) or maintained at similar level in UV6 (SIN, GBS and 4-MGBS) and UV12 (SIN and NGBS) (Figure 8B,C). However, PRO contents in Chinese kale grown under UV6 and UV12 presented decreases of 16.24% and 26.75%, respectively (Figure 8B).

### 2.8. Expressions of Genes of Transcription Factors and Key Enzymes Related to Glucosinolate Biosynthesis Pathway

**Figure 9:**
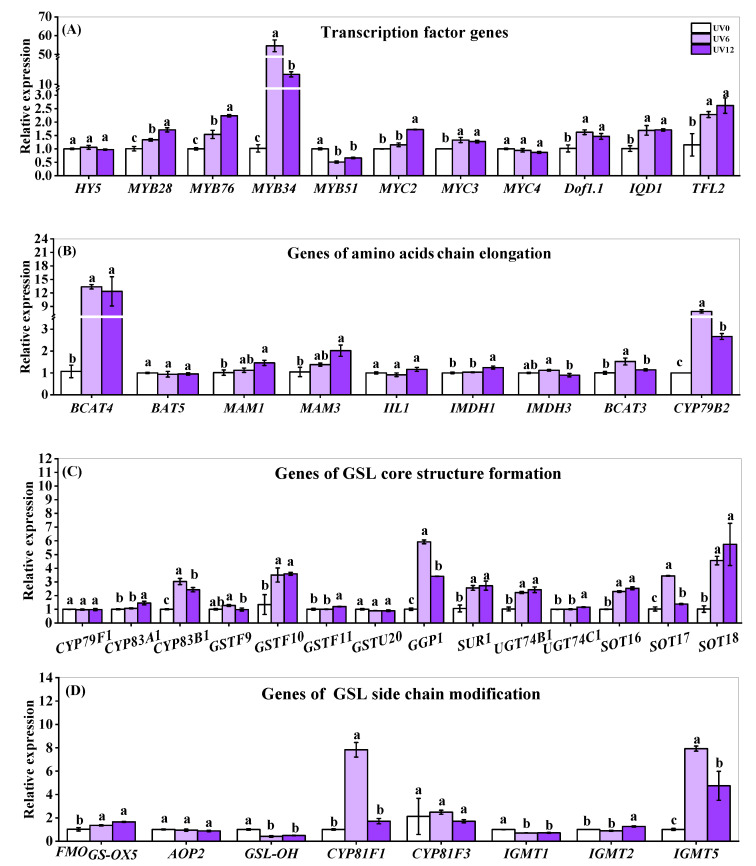
Expressions of genes of transcription factors and key enzymes related to GSL biosynthesis pathways of Chinese kale subjected to different durations of UVA-radiation exposure. (**A**) Transcription factor genes. (**B**) Genes of amino acid chain elongation. (**C**) Genes of GSL core structure formation. (**D**) Genes of GSL side-chain modification. Different lowercase letters (a–c) labeled on the vertical bar (means ± standard error, *n* = 3) represent significant differences by one-way ANOVA with multiple-range tests (*p* ≤ 0.05).

The expressions of genes encoding key enzymes responsible for GSL biosynthesis and related transcription factors (TFs) were monitored (Figure 9). Nine TFs involved in the regulation of GSL biosynthesis changed differently with apparent effects of UVA-radiation exposure duration (Figure 9A). Most of the TFs (*MYB28*, *MYB76*, *MYB34*, *MYC2*, *MYC3*, *Dof1.1*, *IQD1* and *TFL2*) were significantly up-regulated by UV6 and UV12, while *MYB51* was down-regulated. Among all differentially expressed TFs genes, *MYB28*, *MYB76* and *MYC2* were more sensitive to more prolonged UVA-radiation exposure (highest expression in plants treated with UV12, followed by UV6), whereas *MYB34* was less sensitive to more prolonged UVA-radiation exposure. However, transcription factor HY5, known as the repressor of MYB TFs of aliphatic GSLs and activator of MYB TFs of indolic GSLs, did not change with the UVA-radiation exposure duration.

In the GSL biosynthesis pathway, many genes were significantly up-regulated by supplemental UVA exposure (Figure 9B–D). Six genes (*BCAT4*, *MAM1*, *MAM3*, *IMDH1*, *BCAT3* and *CYP79B2*) necessary for amino acid chain elongation showed obviously higher expression levels in plants treated with UV6 and/or UV12 than those in UV0-treated plants (Figure 9B). In the GSL core structure formation process, eleven genes (*CYP83A1*, *CYP83B1*, *GSTF10*, *GSTF11*, *GGP1*, *SUR1*, *UGT74B1*, *UGT74C1*, *SOT16*, *SOT17* and *SOT18*) were found to be significantly up-regulated by supplemental UVA exposure (Figure 9C). In the side-chain modification process, in which the individual GSLs were produced, four genes (*FMO_GS-OX5_*, *CYP81F1*, *IGMT2* and *IGMT5*) presented obviously higher expression levels in plants treated with UV6 and/or UV12 (Figure 9C). Inversely, two genes (*GSL-OH* and *IGMT1*) were down-regulated by supplemental UVA exposure (Figure 9D). Among all the differentially expressed GSL biosynthesis genes, *BCAT3*, *CYP79B2*, *CYP83B1*, *GGP1*, *SOT17*, *CYP81F1* and *IGMT5* showed the highest expression in plants treated with UV6, followed by UV12 (Figure 9B–D).

### 2.9. Gene Expressions of Photoreceptors

**Figure 10:**
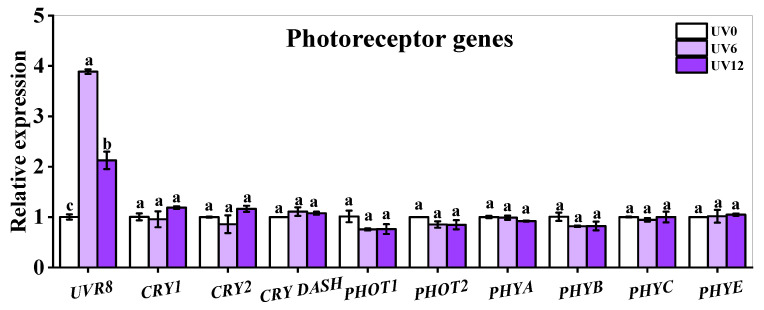
Expressions of photoreceptor genes of Chinese kale subjected to different durations of UVA-radiation exposure. Different lowercase letters (a–c) labeled on the vertical bar (means ± standard error, *n* = 3) represent significant differences by one-way ANOVA with multiple-range tests (*p* ≤ 0.05).

The expressions of genes encoding photoreceptors (UVR8, CRYs, PHOTs and PHYs) were analyzed (Figure 10). Most of the detectable photoreceptor genes (*CRY1*, *CRY2*, *PHOT1*, *PHOT2*, *PHYA*, *PHYB*, *PHYC* and *PHYE*) showed no significant changes in Chinese kale treated with supplemental UVA exposure, except for UVB receptor genes *UVR8*, which was up-regulated. Besides, *UVR8* showed the highest expression in plants treated with UV6, followed by UV12 (Figure 10).

### 2.10. PCA and Heatmap

**Figure 11:**
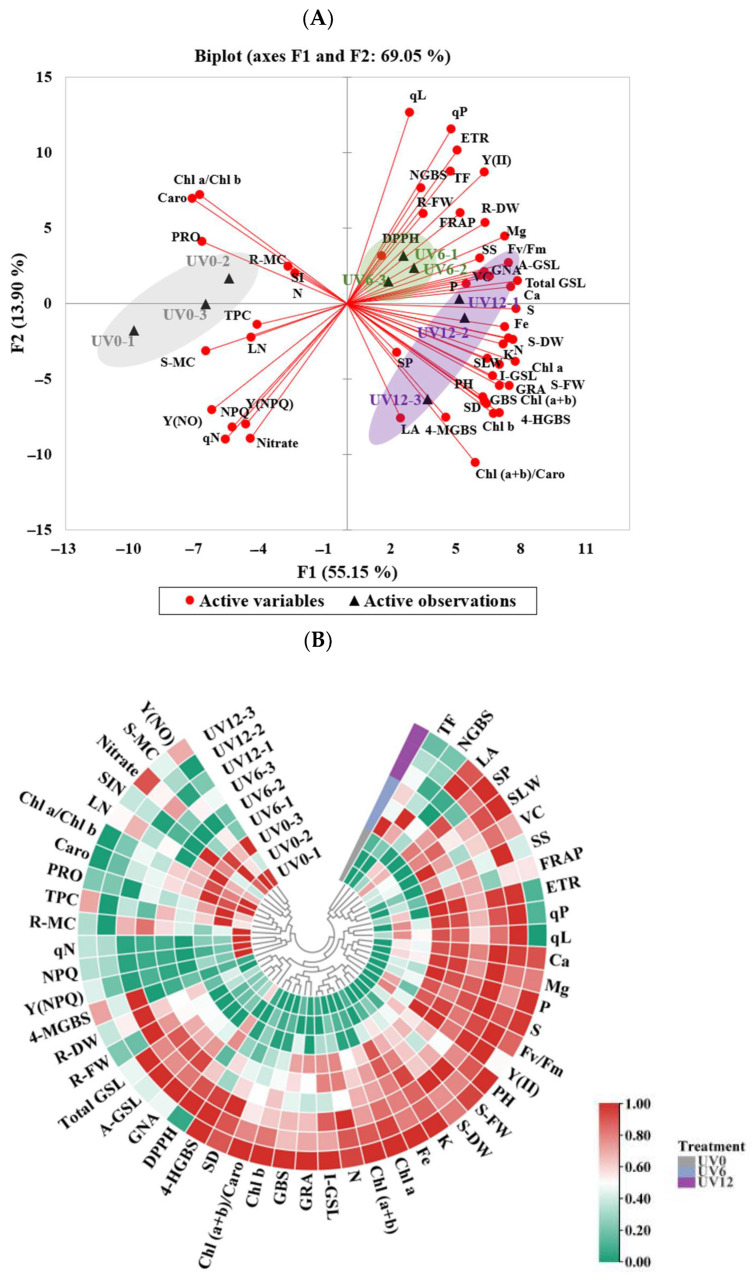
Principal component (**A**) and heatmap (**B**) analyses showed differences and correlations in the investigated parameters in Chinese kale subjected to different durations of UVA-radiation exposure. Results are visualized using a false-color scale, with red and green indicating an increase and a decrease in the response parameters.

A PCA and a heatmap were used to visualize the effects of different UVA-radiation exposure durations on the growth and the phytochemical composition of Chinese kale (Figure 11). Overall, PC1 and PC2 explained 55.15% and 13.90%, respectively (Figure 11A). The treatments were divided into three clear groups in the PCA scatter plot, with the UV0, UV6 and UV12 treatments being distributed in distinct quadrants. Chinese kale plants in the right quadrants (subjected to UV6 and UV12 treatments) were characterized by higher agronomic performance and nutritional composition.

The parameters could be grouped into three clusters across the heatmap (Figure 11B), corresponding to the durations of supplemental UVA exposure. The hierarchical clustering analysis showed that the UV6 and the UV12 clusters were the closest to each other in the measured parameter responses. Higher biomass and phytochemical contents were observed in these two clusters. The UV0 cluster was separated from the other two clusters due to its lower biomass and phytochemical contents.

## 3. Discussion

### 3.1. Prolonging UVA-Radiation Exposure Accelerated the Growth of Chinese Kale

The response of plants to UVA radiation has recently received extensive attention. There is currently no consensus on the effect of supplemental UVA exposure on plant growth. Previously, supplemental UVA exposure inhibited the growth of wheat, cotton and sorghum [19]. However, UVA-radiation exposure had a positive effect on the growth of Chinese kale baby leaf [11], kale [20,21] and tomato seedling [22]. In these studies, this positive effect displayed a saturation response to the UVA dose. Supplementing UVA (369 nm) to PAR in a controlled environment prominently accelerated the growth of tomato seedlings, causing 29% and 33% increases in plant dry weights when exposed to supplemental UVA exposure for 8 h/d and 16 h/d, respectively, and there were no significant differences between them [22]. Moreover, 5, 10 and 15 days of UVA exposure resulted in −18%, −32% and −30% higher shoot dry weights, and there were no significant differences between 10 and 15 days of UVA exposure treatments [6].

In this study, supplemental UVA treatments on 25-day-old Chinese kale were carried out for 20 days. The fresh and the dry weights of Chinese kale shoots were enhanced, but the moisture content was reduced by the increased duration of supplemental UVA exposure (Figure 1), suggesting that the increase in fresh weight was mainly due to the increase in dry weight. Moreover, prolonged UVA-radiation exposure increased plant height, stem diameter and specific leaf weight, with unchanged leaf number and leaf area (Figure 2). The increase in leaf thickness might be due to the fact that UVA-radiation exposure could increase the thickness of palisade parenchyma and dorsal epidermis, but not the thickness of spongy tissue and adaxial epidermis [23]. The increase in leaf thickness might have decreased UVA penetration within leaves, increased photosynthesis and thus accumulated dry weight.

With the increase in the duration of UVA-radiation exposure, chlorophyll a/b in Chinese kale plants showed a decreasing trend, and chlorophyll b content increased more than chlorophyll a content (Figure 3E). More chlorophyll b, as a part of the light-trapping pigment–protein complex, was produced, which might have helped the plant to receive more UVA, with the increase in the duration of UVA exposure. Supplemental UVA treatments significantly promoted chlorophyll synthesis, whereas they significantly reduced carotenoid content, and the effects were more significant with the increase in supplemental UVA exposure duration in this study (Figure 3A–D). Similarly, UVA supplementation promoted chlorophyll synthesis and inhibited carotenoid synthesis in two lettuce varieties, ‘Red butter’ and ‘Yanzhi’ [24].

Accelerating plant uptake of mineral elements is vital to increase plant growth [25]. N and Mg are components of chlorophylls, while Fe plays a catalytic role in chlorophyll biosynthesis. In this study, the accumulations of these elements (N, Mg and Fe), as well as the chlorophyll contents (Figure 3A–C), in Chinese kale significantly increased in plants subjected to supplemental UVA treatments (UV6 and UV12) (Figure 6). In addition, N accumulation (Figure 6), chlorophyll contents (Figure 3A–C) and shoot biomass (Figure 1A,B) in Chinese kale increased with the increase in supplemental UVA exposure duration. The accumulation of P, K, Ca, Mg, S and Fe significantly increased in plants subjected to UV6 and UV12 treatments, whereas there were no significant differences between UV6 and UV12. As also shown in Figure 11A, the relationships among the different variables (i.e., shoot dry weight and phytochemical contents), where two vectors formed an angle smaller than 90°, were positively correlated. The clustered heatmap confirmed the PCA results (Figure 11B). Chinese kale plants in the right quadrants of the PCA (subjected to UV6 and UV12 treatments) were characterized by higher shoot dry weights, and the shoot dry weights showed a strong positive correlation with N accumulation (0.945) and chlorophyll contents (0.820–0.888), whereas N accumulation displayed a strong positive correlation with chlorophyll contents (0.838–0.923) (Table S2). Therefore, the shoot biomass of Chinese kale subjected to UV12 treatment was higher than that of plants subjected to UV6 treatment, which might mainly be because the prolonged UVA-radiation exposure accelerated the N uptake and promoted chlorophylls synthesis.

In addition, the flower budding rate of Chinese kale plants 45 days after being sown increased with the increase in UVA supplemental exposure, reaching 32%, 45% and 66%, respectively (Figure 2F). However, 0.4, 0.6 and 0.8 W/m^2^ UVA intensities supplemented with red and blue light (200 μmol/m^2^/s) had little effect on stem dry weight and diameter of tomato seedlings, and irradiation with UVA (368 nm) for 1 h/d induced flowering earlier than plants treated for 4 h/d [26]. So, UVA exposure could affect plant growth, which depended on plant variety and UVA-radiation exposure duration.

Any stress can lead to the inactivation of photosystem II (PSII) or to a continuous quenching that reduces the maximum quantum efficiency of photosystem II (PSII) for photochemistry (Fv/Fm). The chlorophyll content (SPAD value) in tomato leaves decreased with the increase in supplemental UVA intensity [26], which might be because UVA radiation damaged the chloroplast structure of tomato leaves and affected chlorophyll biosynthesis, which was consistent with UVB damage [27]. The wavelength of UVA is usually shorter and more energetic than that of blue light. Therefore, when irradiated by UVA, seedlings received more light stress, which decreased the Fv/Fm values [26]. However, in the present study, the Fv/Fm values of Chinese kale plants were significantly increased by adding UVA (40 μmol/m^2^/s) to PAR at 250 μmol/m^2^/s (Figure 4A). It could be inferred that supplemental UVA exposure benefitted the photosynthesis of Chinese kale.

Y(II) was significantly increased by UVA irradiation, indicating that UVA exposure could improve the actual photochemical efficiency of PSII under illumination (Figure 4B). Y(NO) and Y(NPQ) are important fluorescence parameters of light damage and light protection, respectively [28]. In this study, supplemental UVA treatments had little effect on Y(NO), but UV6 significantly weakened the photoprotection ability (measured as Y(NPQ)) of Chinese kale. NPQ and qN are non-photochemical quenching indexes. NPQ did not change significantly in plants treated with UV6 and UV12, indicating that UVA-radiation exposure left unchanged the thermal dissipation of excess energy in Chinese kale. However, qN significantly decreased after UV6 treatment, indicating that UVA treatment for 6 h/d reduced heat dissipation but increased fluorescence and the potential photochemical efficiency. It was previously reported that UVA supplementation at unsaturated visible light levels could increase the photosynthetic rate, mainly due to UV-induced violet–blue–green fluorescence, which was collected by photosynthetic pigments to drive electron transport [29]. However, in this study, the electron transfer rate (ETR) was not affected by supplementary UVA exposure. These findings need more research to be explained.

### 3.2. UVA Radiation Administered for Different Durations Affected the Phytochemical Contents of Chinese Kale

UVA supplementation treatments (6 h/d and 12 h/d) significantly increased the soluble sugar content of Chinese kale (about 35.00%) (Figure 5B). Supplementation with UVA (10, 20 and 30 μmol/m^2^/s for 16 h/d) promoted the accumulation of soluble sugar (12.74–26.11%) in ‘Klee’ lettuce, and treatment with 10 μmol/m^2^/s UVA was the most effective [6]. Supplementation with UVA (10 μmol/m^2^/s, 10 h/d) increased the soluble sugar content (13.29%) in ‘Red butter’ lettuce [24]. Higher Fv/Fm values meant stronger photosynthesis and sugar transport in plants [30]. Compared with UV0, the Fv/Fm values and soluble sugar contents of Chinese kale treated with UVA were higher (Figure 4A and Figure 5B), and their positive correlations were high (0.735) (Table S2). UVA exposure affected nitrate uptake and promoted nitrate–nitrogen metabolism [10]. In this study, the nitrate content of UV6-treated Chinese kale was significantly reduced; however, that of UV12-treated Chinese kale did not decrease (Figure 5C). The nitrate content of lettuce increased with UVA supplemented before dark [31]. Plant nitrate content might respond differently to pre-dark, light and post-dark UVA supplementations.

Antioxidants such as vitamin C, flavonoids and phenolic compounds significantly contribute to the total antioxidant capacity [10]. DPPH and FRAP are commonly used to assess the antioxidant capacities of vegetables. UVA (380 nm) supplementation treatment significantly increased DPPH and FRAP in red- and green-leaf pak-choi [10] and significantly increased DPPH and FRAP in ‘Green butter’ lettuce [31]. UVA (380 nm) supplementation treatment significantly increased DPPH in ‘Yanzhi’ lettuce but had no significant effects on FRAP [24]. UVA (380 nm) supplementation treatment significantly decreased DPPH but not FRAP in ‘Red butter’ lettuce [31]. In the present study, UVA supplementation had no effects on DPPH, but UV6 treatment significantly increased FRAP (Figure 5G,H). Moreover, the trend of FRAP was consistent with the trend of total flavonoid content obtained with all treatments (Figure 5E and Figure 11A). The relationship (0.816) between the total antioxidant activity (FRAP) and the total flavonoid content in Chinese kale leaves exposed to supplementary UVA is shown in Table S2. UVA irradiation on plants might cause plants to develop various protective response mechanisms, such as the accumulation of antioxidants, which act to inhibit oxidative damage. UVA-induced flavonoids could shield plants from UVA to prevent UVA-induced cell damage [32]. Kale treated with two UVA LEDs (370 and 385 nm) at 30 W/m^2^ for 5 d and Chinese kale treated with two supplemental UVA-radiation durations (6 h/d and 12 h/d) at 40 μmol/m^2^/s for 20 d exhibited no morphological damage [21], indicating that these UVA wavelengths did not cause permanent oxidative damage to plants.

A UVA/PAR ratio of 4.35% (i.e., UVA of 10 μmol/m^2^/s) greatly improved the yield and quality of lettuce, but further increases to 8.7% (i.e., UVA of 20 μmol/m^2^/s) and 13.05% (i.e., UVA of 30 μmol/m^2^/s) did not have better results [6], probably because lettuce cultivation in a controlled environment does not require a higher than natural UVA/PAR ratio (7–8%, measured in Beijing). However, in this study, UVA supplementation treatment was administered at 40 μmol/m^2^/s, which greatly improved the yield and quality of Chinese kale (Table 1 and Figure 11A), so a higher UVA/PAR ratio than natural sunlight might be required for Chinese kale cultivation. In addition, UVA treatment increased the contents of chlorophylls, soluble sugars and flavonoids and did not have a concentrated effect, because the biomass of Chinese kale increased, and similar results were observed in kale under supplemental UVA-radiation exposure [21]. The biomass of ‘Green butter’ lettuce [31] and ‘Yanzhi’ lettuce [24] did not change when exposed to UVA but instead promoted the accumulation of various bioactive compounds. Thus, UVA irradiation has the potential to promote plant growth and the accumulation of bioactive compounds. This might be achieved through the allocation of additional carbohydrates due to the enhanced photosynthesis caused by UVA supplementation, which stimulates biomass and secondary metabolite accumulation [33].

### 3.3. Supplemental UVA-Radiation Exposure Stimulated Glucosinolate Accumulation by Promoting the Expressions of Genes Involved in Glucosinolate Biosynthesis of Chinese Kale

In this study, eight individual GSLs were identified and quantified in Chinese kale using HPLC (Figure 7). Supplemental UVA exposure caused a significant increase in GSL contents, including the total aliphatic GSLs, total indolic GSLs, total GSLs and most of the individual GSLs (except PRO among the eight GSLs) (Figure 8). Similarly, supplemental UVA (400 nm) exposure led to a noticeable increase in total aliphatic GSLs and total GSLs in green-leaf pak-choi baby leaf [11], and UVA (380 nm and 400 nm) exposure led to a marked increase in total aliphatic GSLs, total indolic GSLs and total GSLs in Chinese kale baby leaf [11] and red-leaf pak-choi [10]. Supplemental UVA (380 nm) exposure displayed a significant positive effect on the contents of total aliphatic GSLs and total GSLs in Chinese kale baby leaf [34]. In broccoli, among glucosinolates, only GBS was increased by supplemental UVA (365 nm) exposure [13], while only 4-MGBS showed a 2-fold increase after exposure to UVA (365 nm) in combination with less blue and more red background spectrum [14]. However, the contents of PRO, SIN and GNA in green-leaf pak-choi baby leaf were dramatically affected by supplemental UVA (380 nm) exposure [11].

The expressions of genes encoding key enzymes involved in GSL biosynthesis (Figure 9B–D), GSL-related TFs (Figure 9A) and the light-signaling-related photoreceptors (Figure 10) were determined. Supplemental UVA exposure could up-regulate the expressions of the majority of GSL biosynthesis-related genes in chain elongation (*MAM3*, *IMDH1*, *BCAT3* and *CYP79B2*) and core structure formation (*CYP83A1*, *CYP83B1*, *GSTF10*, *GSTF11*, *GGP1*, *SUR1*, *UGT74B1*, *UGT74C1*, *SOT16*, *SOT17* and *SOT18*), as well as side-chain modification (*FMO_GS-OX5_*, *CYP81F1*, *IGMT2* and *IGMT5*) (Figure 9B–D). Five genes (*CYP79B2*, *CYP83B1*, *GGP1*, *SOT17* and *IGMT5*) showed significantly higher expressions in UV6-treated than in UV12-treated plants; besides, only UV6 led to the up-regulated expressions of two genes (*BCAT3* and *CYP81F1*), and only UV12 led to the up-regulated expressions of six genes (*MAM3*, *IMDH1*, *CYP83A1*, *GSTF11*, *UGT74C1* and *IGMT2*) (Figure 9B–D). These indicated that UVA-regulated expressions of genes involved in GSL biosynthesis might have signal and dose effects.

The aliphatic GSL transcriptional activators (*MYB28* and *MYB76*), indolic GSL activator *MYB34* and other transcriptional activators (*MYC2*, *MYC3*, *Dof1.1*, *IQD1* and *TFL2*) were up-regulated by supplemental UVA exposure (Figure 9A). In contrast, the inversely down-regulated *MYB51* (aliphatic activator) added to the complexity of the TF-regulated GSL biosynthesis network. Though the aliphatic GSL transcriptional activators (*MYB28* and *MYB76*) showed significantly higher expressions in UV12-treated than in UV6-treated plants, the contents of total aliphatic GSLs and individual aliphatic GSLs showed no differences (Figure 8A,B). However, indolic GSL activator *MYB34* showed significantly higher expression in UV6-treated than in UV12-treated plants, and the contents of individual indolic GSLs were similarly affected by the duration of supplemental UVA exposure (Figure 8C).

The expressions of most photoreceptors were unchanged, but *UVR8* was up-regulated by UV6 and UV12 (Figure 10). UVA-radiation can effectively induce cryptochrome (CRY) and phototropin (PHOT) signal transduction [35]. In *Arabidopsis thaliana*, UVR8 is generally activated by UVB (280–315 nm) radiation, and it also responds to UVA (365 nm, 15 μmol/m^2^/s) radiation and promotes the production of antioxidants [36]. Additionally, *UVR8* presented UVA-dose dependency, with the most apparent up-regulation obtained with UV6 treatment, as well as a relatively tender but also significant up-regulation caused by UV12 (Figure 10). Therefore, supplemental UVA exposure might promote the expression of *UVR8*, which might induce an increase in GSLs. However, this speculation needs to be verified by further experiments.

**Figure 12:**
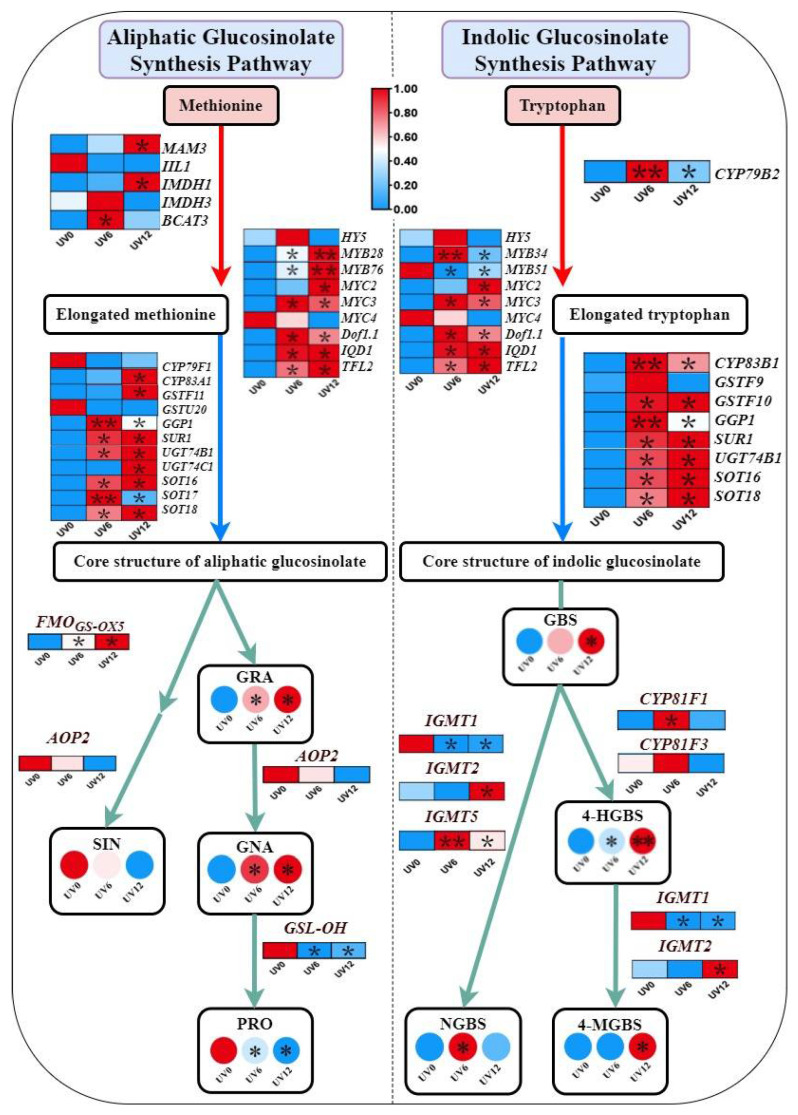
Expression pattern diagram of genes associated with GSL biosynthesis in plants subjected to different UVA-radiation exposure durations. The red arrows indicate amino acid chain extension, while the blue arrows indicate glucosinolate core structure formation, and the green arrows indicate secondary modification of side chains. The rectangles represent genes, and the circles represent glucosinolates. The gene expression levels or the glucosinolate contents ranged from low (blue) to high (red). * and ** indicate that the relative gene expression or glucosinolate content was significantly increased or decreased by UV6 and UV12 compared with UV0 at *p* ≤ 0.05 and at *p* ≤ 0.01, respectively.

The expressions of genes could be presented as a pattern, as shown in Figure 12. With UVA supplementation, the aliphatic transcriptional activators (*MYB28* and *MYB76*), an indolic transcriptional activator (*MYB34*) and other transcriptional activators (*MYC2*, *MYC3*, *Dof1.1*, *IQD1* and *TFL2*) were up-regulated, which contributed to higher expressions of GSL biosynthesis genes in the stages of chain elongation (*MAM3*, *IMDH1*, *BCAT3* and *CYP79B2*) and core structure formation (*CYP83A1*, *CYP83B1*, *GSTF10*, *GSTF11*, *GGP1*, *SUR1*, *UGT74B1*, *UGT74C1*, *SOT16*, *SOT17* and *SOT18*), as well as side-chain modification (*FMO_GS-OX5_*, *CYP81F1*, *IGMT2* and *IGMT5*). Consequently, the GSL contents significantly increased in Chinese kale. Furthermore, the up-regulation observed for *UVR8* indicated that the light-signaling network induced by supplemental UVA might be involved in GSL biosynthesis. In addition, both signal and dose effects were observed in UVA-regulated GSL biosynthesis.

## 4. Materials and Methods

### 4.1. Growth Conditions and Treatments

This study was carried out in the artificial-light plant factory (22 °C, 60% of relative humidity and 565 µmol/mol CO_2_ concentration) of South China Agricultural University (east longitude 113.36°, north latitude 23.16°). Chinese kale (*Brassica alboglabra*, ‘Lvbao’) seeds were directly sown in an 8 cm^3^ sponge block filled with clear water and were placed in a dark germination chamber. After three days, the germinated seeds were put in a deep flow technique (DFT) system with half-strength Hoagland nutrient solution (EC ≈ 1.2 mS·cm^2^, pH ≈ 6.5). Chinese kale plants were cultivated under white 250 μmol/m^2^/s LEDs (Chenghui Equipment Co., Ltd., Guangzhou, China) from 8:00 to 20:00. After twelve days, the three-true-leaf seedlings were transplanted to the DFT system with nutrient solution (944 mg/L Ca(NO_3_)_2_·4H_2_O; 404 mg/L KNO_3_; 160 mg/L NH_4_NO_3_; 200 mg/L KH_2_PO_4_; 348 mg/L K_2_SO_4_; 492 mg/L MgSO_4_·7H_2_O, EC ≈ 2.0 mS·cm^2^, pH ≈ 6.0) at the density of 24 plants/planting plate (90 cm × 60 cm).

**Figure 13:**
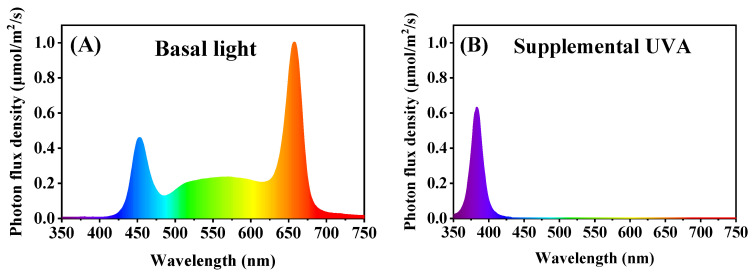
Light spectra of basal light (**A**) and supplemental UVA (**B**).

Ten days after transplantation, the plants were treated with a supplemental UVA LED (380 ± 10 nm, 40 μmol/m^2^/s ≈ 10 W/m^2^, Chenghui Equipment Co., Ltd., Guangzhou, China) for 0 h (UV0), 6 h (UV6) and 12 h (UV12) per day with three replications. The light spectra of basal light and supplemental UVA are shown in Figure 13, and the lighting characteristics of the three light treatments are presented in Table 1.

### 4.2. Growth and Morphology Evaluation

On the 20th day of light treatment, the flower budding rate of Chinese kale plants (45 days of age) was counted for each treatment. Eight uniform Chinese kale plants were randomly selected from each treatment group for the growth characteristics. The sampled plants were cut at the junction of the roots and shoots. The fresh weight (FW) of separated plant parts (g) was weighed using an electronic balance. The plant height (from cotyledon to the apical point; cm) and the stem diameter (between the 4th and 5th true leaves; mm) were measured with a ruler and a 1/1000 digital display vernier caliper. The leaf number was recorded. Then, the shoots and roots were deactivated at 105 °C for 2 h and dried at 70 °C for 72 h to determine the dry weight (DW). Moisture content (MC) (%) = (FW − DW)/FW × 100%. The leaf images were processed with ImageJ 1.46 software (National Institute of Health, USA) to calculate the leaf area (cm^2^). Specific leaf weight (mg/cm^2^) = DW of leaf (mg)/leaf area (cm^2^).

### 4.3. Chlorophyll Fluorescence (ChlF) Property Evaluation

On the 20th day of light treatment, six uniform Chinese kale plants were randomly collected from each treatment group and were moved to a dark room. After 30 min of dark adaptation, the fourth or fifth fully expanded leaves from the top of the plants were measured with the IMAGING PAM modulated fluorometer (MAXI-head) (Walz GmbH, Effeltrich, Germany). Actinic light intensity was set to 81 μmol/m^2^/s of PAR. The maximum quantum yield of PSII (Fv/Fm), the effective quantum yield of PSII (Y(II)), the non-regulated energy dissipation yield of PSII (Y(NO)), the regulated energy dissipation yield of PSII (Y(NPQ)), the non-photochemical quenching (NPQ), the non-photochemical quenching coefficient (qN), the photochemical quenching (qP), the fraction of open PSII centers (qL) and the electron transport rate (ETR) were measured and recorded.

### 4.4. Pigment Content Evaluation

On the 20th day of light treatment, nine uniform Chinese kale plants were randomly collected from each treatment group (3 plants/replicate, 3 replicates/treatment). Fresh Chinese kale leaves (0.5 g) were soaked in 20 mL of 95% ethanol in the dark until they turned white. Then, the extraction absorbance was measured using a UV spectrophotometer (Shimadzu UV-16A; Shimadzu, Corporation, Kyoto, Japan) at 665 nm (A_665_), 649 nm (A_649_) and 470 nm (A_470_). The pigment contents were calculated according to Lichtenthaler [13] as follows: chlorophyll (Chl) a content (mg/g FW) = (13.36 × A_665_ − 5.19 × A_649_) × 20 mL/(1000 × 0.5 g); Chl b content (mg/g FW) = (27.43 × A_649_ − 8.12 × A_665_) × 20 mL/(1000 × 0.5 g); Chl (a + b) content (mg/g FW) = (5.24 × A_665_ + 22.24 × A_649_) × 20 mL/(1000 × 0.5 g); carotenoid (Caro) content (mg/g FW) = (4.78 × A_470_ − 3.65 × A_665_ − 12.76 × A_649_) × 20 mL/(1000 × 0.5 g).

### 4.5. Shoot Phytochemical Content Evaluation

On the 20th day of light treatment, nine uniform Chinese kale plants were randomly collected from each treatment group (3 plants/replicate, 3 replicates/treatment). The fresh shoots of Chinese kale for phytochemical analyses were sampled and immediately frozen in liquid N_2_, pulverized and stored at −80 °C until analysis.

The soluble protein (SP) content was measured with the Coomassie brilliant blue G250 dye method [37]. The sample (0.5 g) was mixed well in distilled water (8 mL), and the mixture was centrifuged at 3000 rpm for 10 min. The supernatant (0.2 mL) was homogenized in distilled water (0.8 mL) and Coomassie brilliant blue G250 solution (0.1 g/L, 5 mL). After homogenizing, soluble protein solution was detected at 595 nm using a UV spectrophotometer (Shimadzu UV-16A; Shimadzu, Corporation, Kyoto, Japan).

The soluble sugar (SS) content was evaluated by anthrone colorimetry [38]. The sample (0.5 g) was mixed with 10 mL of distilled water and heated in a boiling water bath for 30 min. The mixture was centrifuged at 3000 rpm for 10 min, and the supernatant (0.1 mL) was mixed with distilled water (1.9 mL), anthrone ethyl acetate (0.5 mL) and vitriol (5 mL). After mixing well, soluble sugar solution was measured at 630 nm using a UV spectrophotometer (Shimadzu UV-16A; Shimadzu, Corporation, Kyoto, Japan).

The nitrate content was determined by salicylic sulfuric acid colorimetry [39]. The sample (1.0 g) was homogenized in distilled water (10 mL). The mixture was heated in a boiling water bath for 30 min and was filtered. The filtrate (0.1 mL) was mixed well with 5% salicylic sulfuric acid (0.4 mL) and 8% sodium hydroxide (9.5 mL). The nitrate content was detected using a UV spectrophotometer (Shimadzu UV-16A; Shimadzu, Corporation, Kyoto, Japan) at 410 nm.

The vitamin C (VC) content was determined by the method of molybdenum blue spectrophotometry [40], and the sample (0.5 g) was extracted in oxalic acid EDTA solution (25 mL). The homogenate was filtered, and the filtrate (5 mL) was mixed with phosphate–acetic acid (0.5 mL), 5% vitriol (1 mL) and ammonium molybdate (2 mL) in sequence. The absorbance at 705 nm was read after 10 min of mixing the reagents using a UV spectrophotometer (Shimadzu UV-16A; Shimadzu, Corporation, Kyoto, Japan).

The total flavonoid (TF) content was determined using the aluminum nitrate method [41]. The sample (0.5 g) was extracted with 100% ethanol (8 mL) for 30 min at 4 °C. The extract (1 mL) was mixed with 5% sodium nitrite solution (0.7 mL) for 5 min, 10% aluminum nitrate solution (0.7 mL) for 6 min and 5% sodium hydroxide solution (5 mL) for 10 min in sequence. The absorbance at 510 nm was read using a UV spectrophotometer (Shimadzu UV-16A; Shimadzu, Corporation, Kyoto, Japan).

The total phenolic compound (TPC) content was determined using the method of Folin–Ciocalteu colorimetry [42]. The sample (0.5 g) was extracted with absolute ethyl alcohol (8 mL) for 30 min. The homogenate was centrifuged at 3000 rpm for 10 min at 4 °C. The supernatant (1 mL) was mixed well with twice-diluted Folin phenol (0.5 mL), 26.7% sodium carbonate solution (1.5 mL) and distilled water (7 mL). After reacting for 2 h, the absorbance at 760 nm was read using a UV spectrophotometer (Shimadzu UV-16A; Shimadzu, Corporation, Kyoto, Japan).

The 2,2-diphenyl-1-picrylhydrazyl (DPPH) radical scavenging rate was measured according to Tadolini et al. [43]. The sample (0.5 g) was extracted with 100% ethanol (8 mL) for 30 min. The mixture was centrifuged at 3000 rpm for 10 min at 4 °C. The supernatant (2 mL) was mixed well with 0.2 μmol/L DPPH solution (2 mL), and the radical scavenging rate was determined using a UV spectrophotometer (Shimadzu UV-16A; Shimadzu, Corporation, Kyoto, Japan) at 517 nm.

The ferric-reducing antioxidant power (FRAP) was analyzed as previously described [44]. The sample (0.5 g) was extracted with 100% ethanol (8 mL) for 30 min at 4 °C. The mixture was centrifuged at 3000 rpm for 10 min. The supernatant (0.4 mL) was homogenized in 0.3 mol/L acetate buffer (3 mL), 10 mmol/L 2, 4, 6-tripyridyl-S-triazine (0.3 mL) and 20 mmol/L ferric trichloride (0.3 mL) for 10 min at 37 °C. The absorption was determined at 593 nm using a UV spectrophotometer (Shimadzu UV-16A; Shimadzu, Corporation, Kyoto, Japan).

### 4.6. Mineral Element Determination

The dried shoot sample was weighed and smashed. The contents of total nitrogen (N), phosphorus (P) and potassium (K) were determined using Ojeda’s method [45], vanadate–molybdate yellow colorimetry [46] and flame photometry method [47], respectively. The contents of calcium (Ca), magnesium (Mg), sulfur (S) and iron (Fe) were calculated by atomic absorption spectrophotometry [48]. Mineral element accumulation (mg/shoot DW) = Mineral element content (mg/g) × shoot DW (g).

### 4.7. Glucosinolate Determination, Identification and Quantification

GSLs were extracted and analyzed as previously described [49]. The sample was freeze-dried by a freeze-drying system (Labconco FreeZone 4.5L, USA) and then pulverized (FW100, China). The dried and pulverized sample (0.2 g) was extracted with 70% methanol (4 mL) in a 75 °C water bath for 20 min, shaking 2–3 times every 5 min. Then, 0.4 mol/L barium acetate (2 mL) was added and well shaken. After 5 min, the extracts were centrifuged at 4000 rpm for 10 min, and the supernatants were collected to flow through the chromatographic columns filled with DEAE-Sephadex A25, then mixed with 0.5 mg/mL sulfatase (0.5 mL) for 10 h. We let the liquid go and eluted desulfoglucosinolates with ultra-pure water (2 mL). Glucotropaeolin (Yuanye Bio-Technology Co., Ltd., Shanghai, China) was used as the internal standard.

The GSLs were separated and analyzed by high-performance liquid chromatography (HPLC; Waters Alliance e2695). HPLC conditions: column, C18 (5 μm, 250 mm × 4.6 mm; Waters, Milford, MA, USA); solvent system, mobile phase A (ultra-pure water) and mobile phase B (acetonitrile); gradient program, 0–32 min at 0–20% B, 32–38 min at 20% B, 38–40 min at 20–100% B; flow rate, 1.0 mL/min; injection volume, 20 μL; column temperature, 30 °C; UV chromatogram detection wavelength, 229 nm. Individual glucosinolates were identified according to their HPLC retention time and our database. The glucosinolate content was calculated according to the HPLC area and relative response factor (ISO9167-1, 1992).

### 4.8. RNA Extraction and qPCR

On the 10th day of light treatment, nine uniform Chinese kale plants were randomly collected from each treatment (3 plants/replicate, 3 replicates/treatment). Chinese kale leaves were sampled, immediately frozen in liquid N_2_ and stored at −80 °C until RNA extraction. Total RNA was extracted with Eastep Super Total RNA Extraction Kit (Promega Biological Products, Ltd., Shanghai, China) and constructed cDNA with Evo M-MLV RT for PCR Kit (Accurate Biology Co., Ltd., Changsha, Hunan, China). The quantitative real-time PCR (qRT-PCR) reactions were carried out in LightCycler 480 Real-Time PCR equipment (Roche, Basel, Switzerland) with HieffTM qPCR SYBR Green Master Mix Reagent (Yisheng Biotech Co., Ltd., Shanghai, China). The primer sequences of the tested genes of interest according to the *Brassica oleracea* genome are listed in Table S1. The relative gene expression levels were normalized according to *GAPDH* and calculated according to the 2^−ΔΔC^_T_ method [50].

### 4.9. Statistical Analysis

An analysis of variance (ANOVA) was performed using Duncan’s test at *p* < 0.05 with SPSS 26.0 software (Chicago, IL, USA). Column charts were plotted using Origin 2018 software (Origin Lab, Northampton, MA, USA). A principal component analysis (PCA) was carried out with XLStat 2019 software (Addinsoft, New York, NY, USA). The heatmap was constructed with TBtools [51].

## 5. Conclusions

In summary, the growth and the phytochemicals of Chinese kale were affected by different durations of UVA supplementation. UVA irradiation for 12 h/d was more beneficial for the growth of Chinese kale, and UVA irradiation for 6 h/d was more beneficial for improving the nutritional quality of Chinese kale. UVA supplementation could promote the biosynthesis of beneficial glucosinolates such as GRA and GBS; it might up-regulate the expression levels of light receptor gene *UVR8* and transcription factor genes *MYB28*, *MYB76* and *MYB34* and induce the expressions of key genes in the glucosinolates biosynthesis pathway. UVA supplementation down-regulated the expression of side-chain modification gene *GSL-OH* and reduced the content of harmful glucosinolate PRO. This study provides a reference for the optimization of the light environment of Chinese kale cultivation in plant factories and provides a reference for the mechanism of UVA affecting the synthesis and regulation of glucosinolates in cruciferous vegetables.

## Figures and Tables

**Table 1:** The lighting characteristics of the three light treatments.

Treatment	UVA-Radiation Duration (h/d)	Supplemental UVA Time of Exposure	UVA (μmol/m^2^/s)	PAR (μmol/m^2^/s)	UVA/PAR (%)
UV0	0	——	40	250	0
UV6	6	08:00–11:00 17:00–20:00	40	250	16
UV12	12	08:00–20:00	40	250	16

## Data Availability

Not applicable.

## Supplementary Materials

The following supporting information can be downloaded at: https://www.mdpi.com/article/10.3390/ijms23147619/s1.
